# An Unusual Case of Penetrating Abdominal Trauma With Important Clinical Lessons

**DOI:** 10.7759/cureus.108927

**Published:** 2026-05-15

**Authors:** Girish Bakhshi, Ranjitha R Maiyya, Chandrakant Sabale, Shweta Tungal, Swapnil Bhagat

**Affiliations:** 1 General Surgery, Grant Government Medical College and Sir JJ Group of Hospitals, Mumbai, IND

**Keywords:** importance of ct in penetrating abdominal injury, intestinal injury by foreign body, occupational hazard around drilling machine, penetrating abdominal trauma, resection anastomosis following through and through intestinal perforation by foreign body

## Abstract

Abdominal trauma is commonly seen nowadays because of increasing road traffic accidents and occupational hazards. This is a case of a 21-year-old patient who suffered a penetrating abdominal injury while working near a drilling machine due to a projectile piece of stone, which caused multiple intestinal perforations with mesenteric injuries. We present a rare case of subacute abdominal pain resulting from a high-velocity, non-missile workplace injury. He underwent an emergency laparotomy within 5 h of presentation and resection and anastomosis with removal of the foreign body. He recovered without sequel and was asymptomatic at three weeks postoperatively. In this study, we want to emphasize the importance of preventive measures to prevent such occupational hazards.

## Introduction

Intestinal injuries, a prevalent consequence of both blunt and penetrating abdominal trauma, demand unwavering attention due to their life-threatening potential [1]. Appropriate evaluation, precise diagnosis, and effective management are paramount to saving lives and mitigating complications. In ancient times, penetrating abdominal injuries were common in wars due to ballistic and explosive injuries, while such injuries may commonly occur in workplaces like mining and road traffic accidents nowadays [2]. In treating abdominal trauma, the mechanism and precise location of injuries serve as vital clues, guiding the clinician’s approach to tailored and effective management [3].

Penetrating traumatic injuries, though less common than blunt trauma, are associated with a higher incidence of intestinal damage. They can be broadly categorized into the following two distinct types: high-energy injuries, such as those caused by projectiles, and low-energy injuries, typically resulting from stab wounds [4]. Explosions in workplaces such as mining and drilling sites can generate high-velocity stone or metal fragments, causing secondary penetrating injuries to nearby workers.

## Case presentation

**Figure 1:**
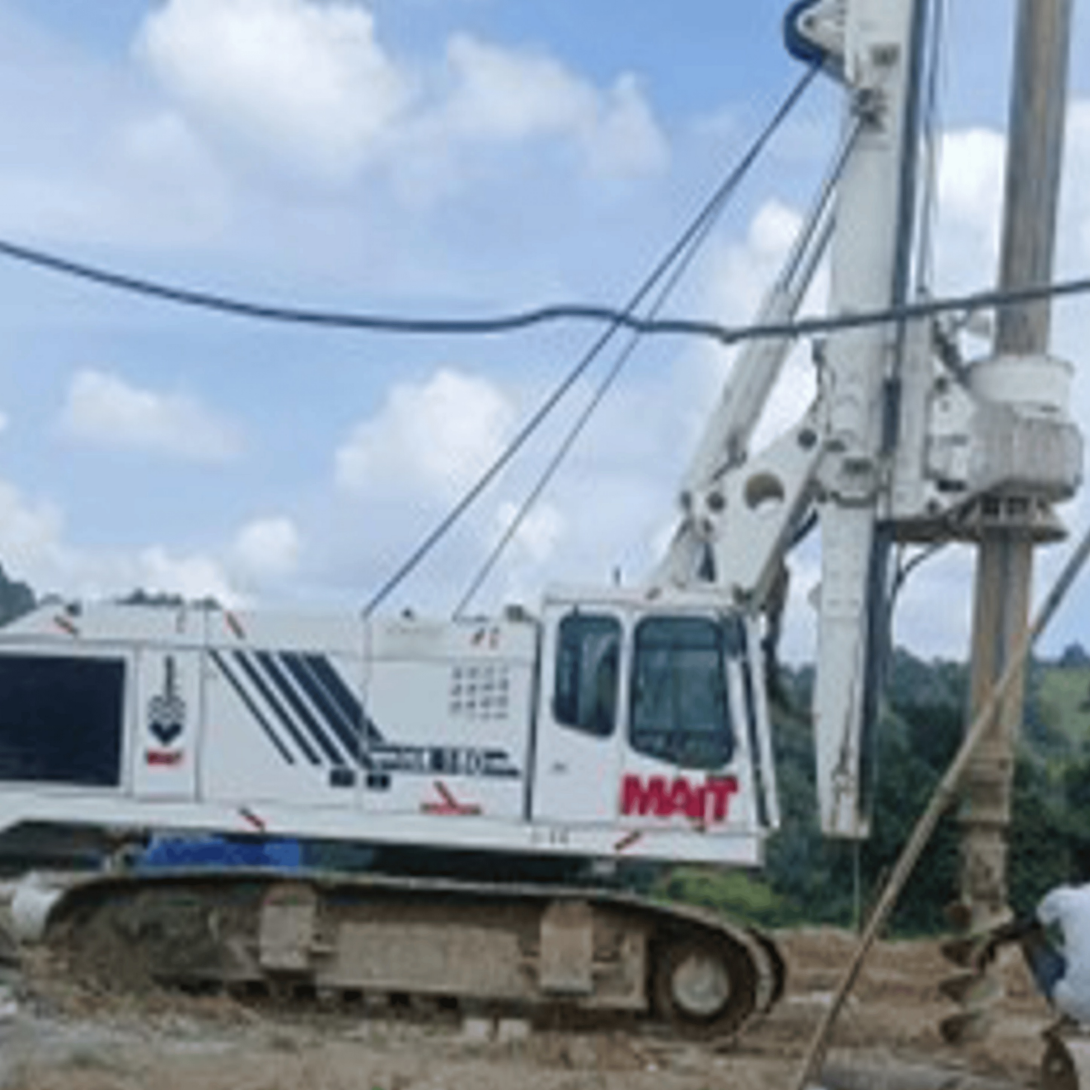
Drilling machine near which the patient was working, causing projection of stone fragments.

A 21-year-old male was brought by his colleagues to the emergency department with an alleged history of injury to the right lower abdomen with a piece of an unknown object projected towards him while working near a drilling machine 2 h back (Figure 1). The object pierced his abdominal wall, causing an ulcer over the right lower abdomen. Following this, the patient developed severe abdominal pain, which progressed rapidly in severity and was aggravated even on slight movement. Initially, he was taken to a private hospital where an X-ray of the pelvis showed a radio-opaque shadow in the pelvis for which the patient was referred to our center.

**Figure 2:**
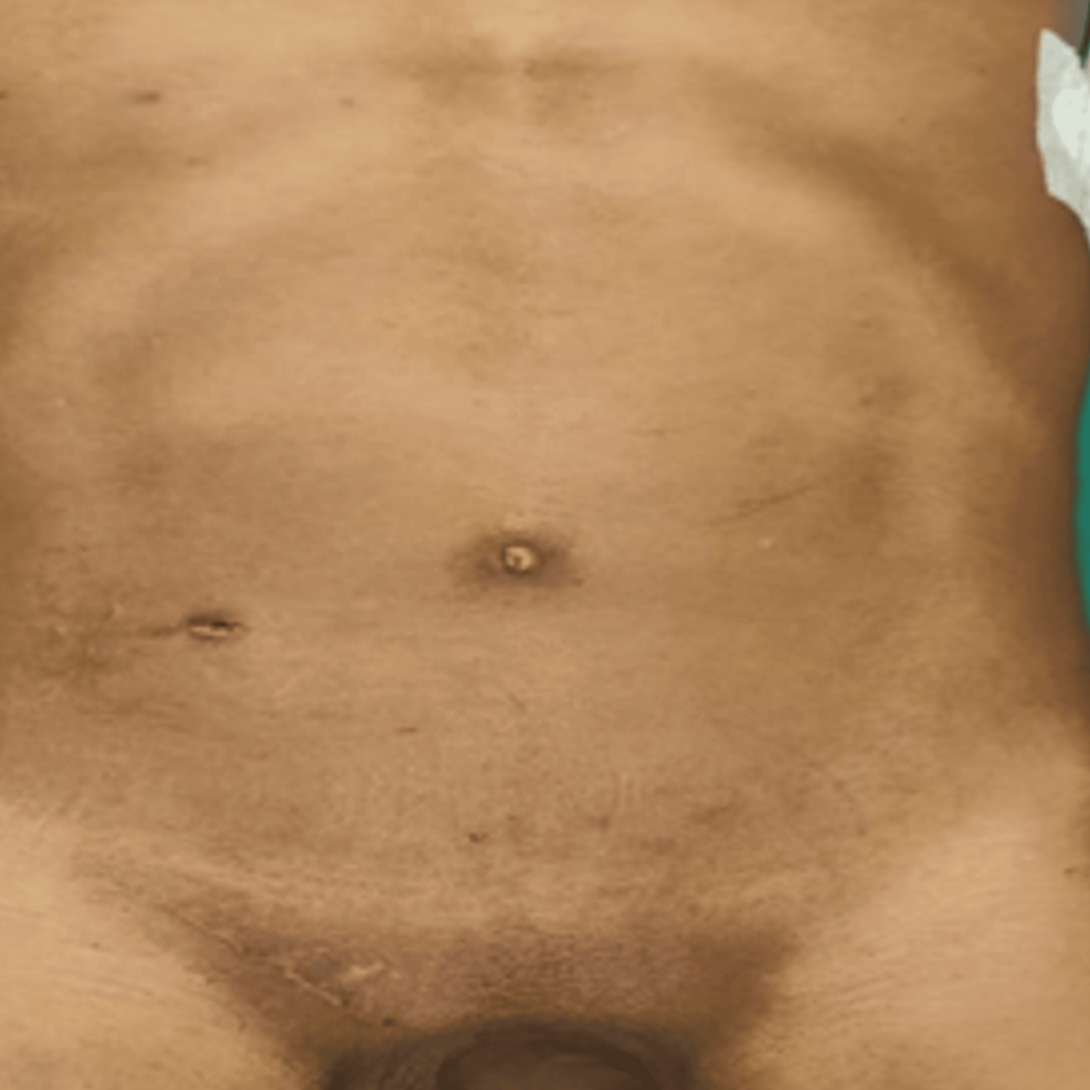
Clinical photo of entry site of projectile stone.

**Figure 3:**
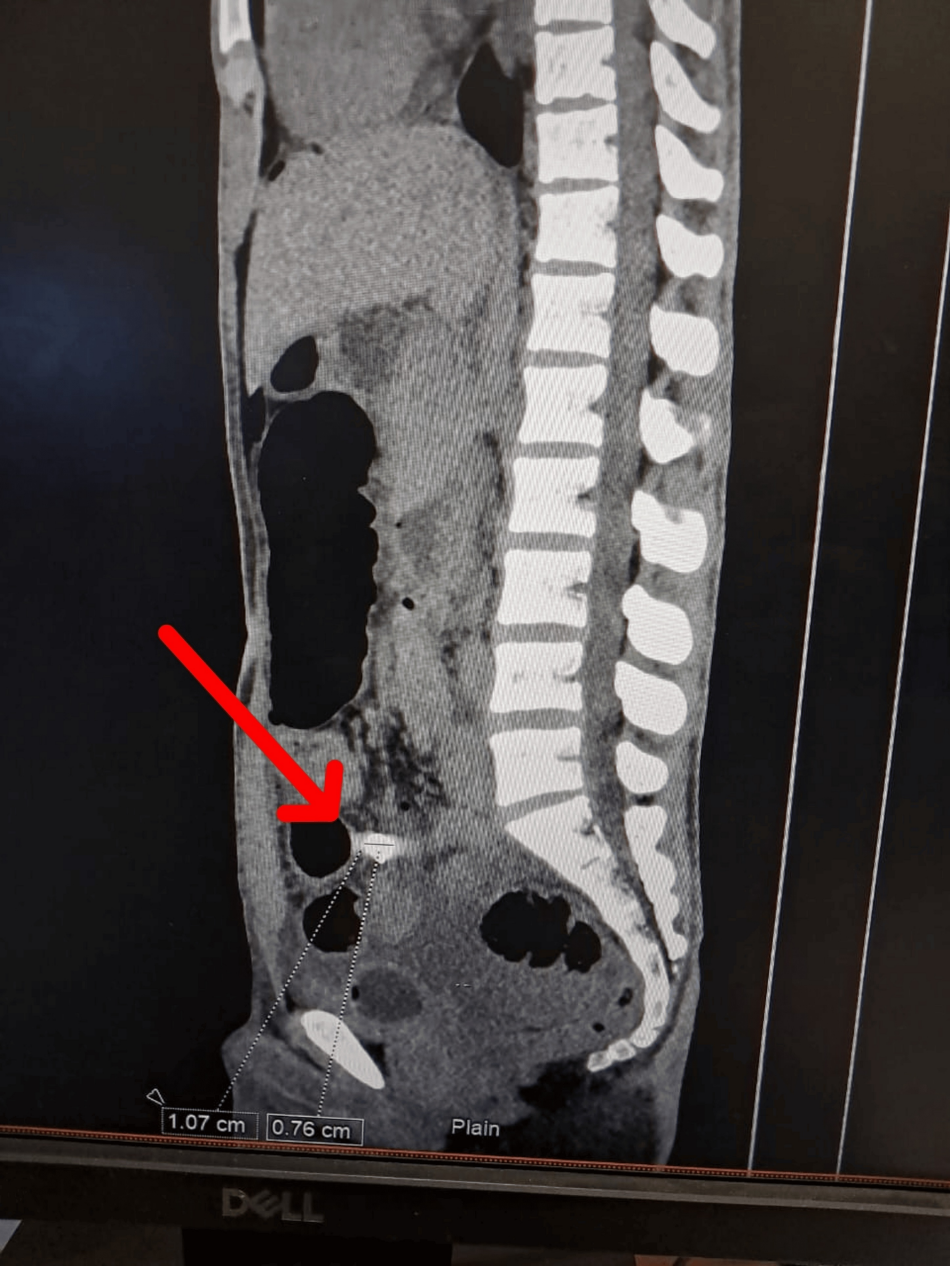
NCCT images localizing the foreign body (arrow). NCCT: non-contrast computed tomography

**Figure 4:**
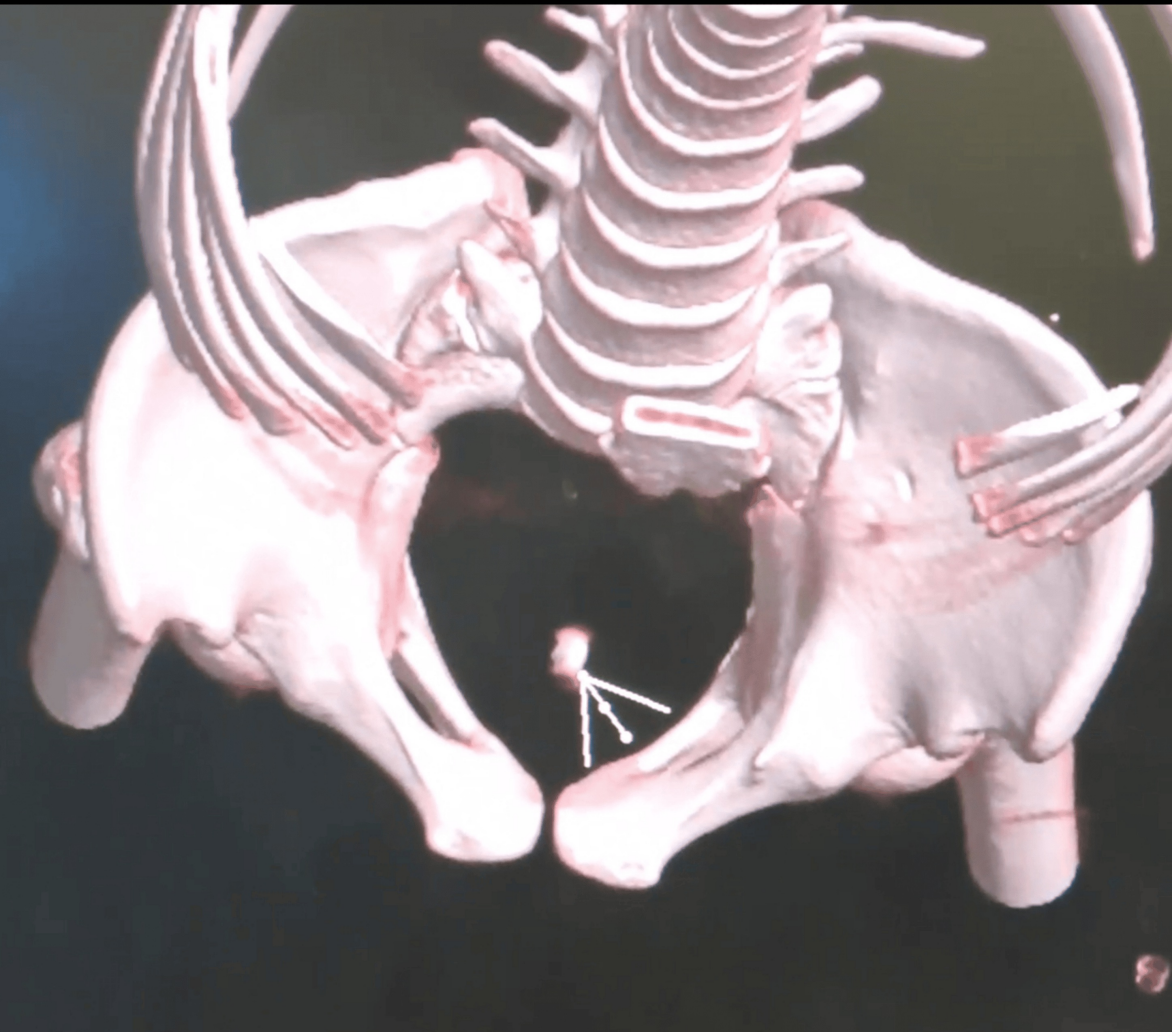
Three-dimensional reconstructed images localizing the foreign body (white lines).

On examination, the patient was conscious, oriented to time, place, and person. He was tachypneic with tachycardia and normal blood pressure. Per abdomen, examination revealed a contused lacerated wound in the right iliac fossa, 2x1 cm in size, without any active bleed/discharge, with diffuse tenderness and guarding (Figure 2). A scar of previously operated right inguinal hernioplasty was noted. Chest X-ray revealed air under the diaphragm. Patient was given IV antibiotics - cephalosporins, analgesics, and hydration. Patient underwent focused abdominal sonography in trauma scan, which revealed moderate hemoperitoneum but could not localize the foreign body. Non-contrast computed tomography scan of abdomen suggested - intra-abdominal foreign body with moderate hemoperitoneum with mild pneumoperitoneum. Patient was then taken to OT for exploratory laparotomy under general anesthesia, after reviewing the radiography and CT findings, within 3 h of presentation (5 h of incident) (Figures 3, 4). The patient was induced under general anesthesia, and the abdomen was opened by a midline incision. Around 150-200 cc of serosanguinous fluid was suctioned from the peritoneal cavity. No blood clots were found in the peritoneal cavity. The ileocecal junction was identified, and the small bowel was traced from the ileocecal junction proximally.

**Figure 5:**
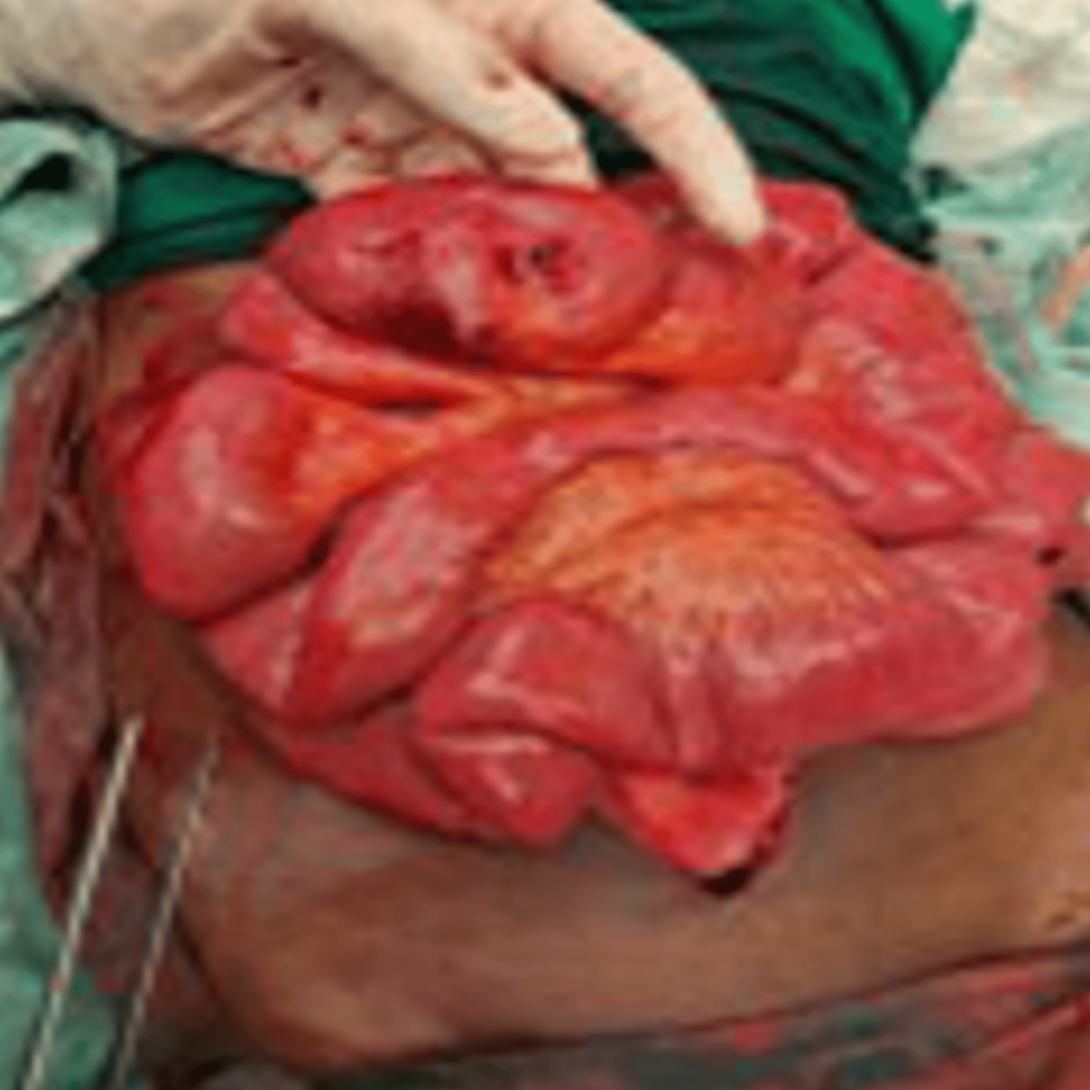
Intra-operative images showing ileal perforation and mesenteric injuries.

**Figure 6:**
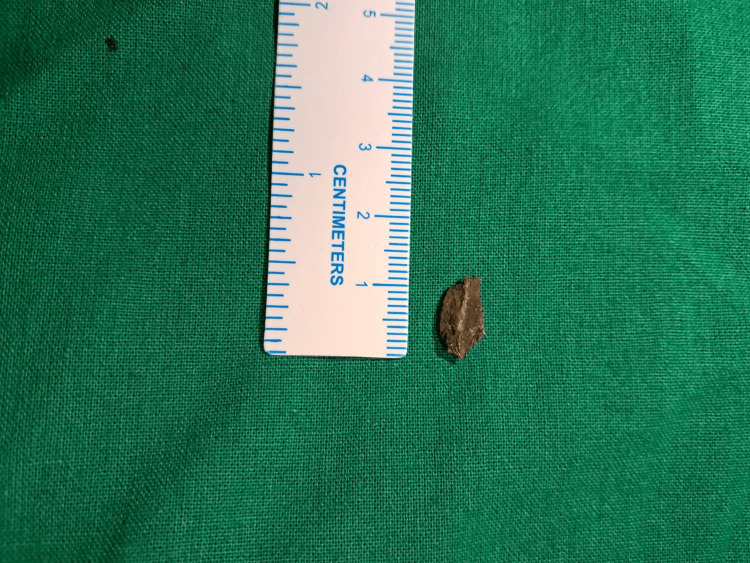
Foreign body that caused intestinal perforation.

Around 50-60 cm from the ileocecal junction, two perforations were identified as seen in the following images given below: a 0.5x0.5 cm perforation near the antimesenteric border, perforating both the walls of bowel, a 1x1 cm perforation at the mesenteric border extending into the mesentery, identified about 4 cm proximal to the first perforation (Figure 5). Approximately 1x0.5x0.3 cm flat, sharp-edged, dark brown to black colored foreign body identified stuck in the mesentery, about 1 cm from the proximal perforation (Figure 6).

**Figure 7:**
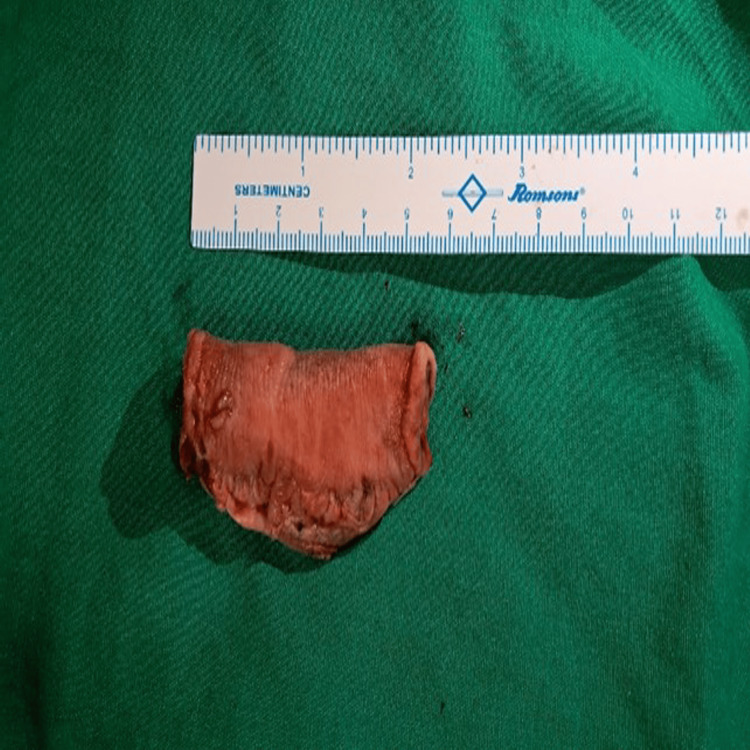
Resected perforated ileal segment.

A 5 cm distal ileal segment, including the two perforations and the site of the foreign body, was resected, and anastomosis of the divided ends was performed (Figure 7). The patient was started on a liquid diet on postoperative day two and tolerated well. The postoperative period was uneventful, and the patient was followed up for a period of four weeks and was asymptomatic.

## Discussion

Gastrointestinal injuries following abdominal trauma are increasingly encountered, largely attributable to the rising incidence of road traffic accidents, and are observed more commonly in young adult males [5]. In earlier times, these types of presentations were common during wars due to gunshots and explosive injuries. Explosives create and energize particles that act as projectiles prone to further fragmentation or create other secondary missiles in the body. Traumatic gastrointestinal perforations predominantly affect the small intestine, with the jejunum being the most frequently involved segment. These injuries are typically managed through primary closure. Penetrating injuries, currently among the most prevalent workplace accidents, are commonly attributed to non-missile, low-velocity sharp objects. However, in the presented case, the causative agent deviates from the norm, being a non-missile, yet high-velocity sharp object, adding an unusual dimension to the injury mechanism. The initial assessment of patients presenting with intestinal trauma should adhere to the standardized trauma evaluation protocol, encompassing a meticulous primary survey to address life-threatening conditions and a subsequent secondary survey to identify specific injuries. Abdominal trauma constitutes a significant proportion of cases encountered in the management of trauma patients. Remarkably, trauma is the leading cause of mortality among individuals under the age of 40 years, underscoring its devastating impact during the most productive years of life. Additionally, it ranks as the third most common cause of death across all age groups, highlighting its universal burden [6].

Bowel injuries, a critical subset of abdominal trauma, may arise from either blunt force impact or penetrating mechanisms. Among these, penetrating abdominal injuries caused by projectiles present a distinctive challenge, often characterized by severe damage due to the high kinetic energy transferred to the tissues. Such injuries demand a vigilant and systematic approach to ensure timely intervention and optimize patient outcomes.

Computed tomography (CT) scanning stands as the gold standard in the diagnosis of intestinal injury in trauma, offering unparalleled sensitivity and specificity. However, in the context of a hemodynamically unstable patient, time is of the essence. In such critical scenarios, imaging should be bypassed in favor of immediate surgical intervention, with the patient being taken directly to the operating room to address life-threatening injuries without delay. In the present case, a computed tomography scan was performed before taking him to surgery to determine the number of foreign bodies, as the patient wasn’t fully aware of how the incident occurred. This scan helps locate foreign bodies and rule out any in the retroperitoneal area that might necessitate another intervention for removal [7].

Intestinal perforation, a serious condition involving a breach in the bowel wall, can arise from trauma, instrumentation, inflammation, infection, malignancy, ischemia, or obstruction. Immediate diagnosis and treatment are crucial to avoid complications like peritonitis and systemic issues due to the leakage of intestinal contents [8]. A detailed history, physical examination, and supportive tests are essential for timely diagnosis and effective management. Prompt intervention is key to reducing morbidity and mortality. There are ultimately the following four mechanisms that can lead to a perforation of the intestinal tract: ischemia (bowel obstruction, necrosis), infection (appendicitis, diverticulitis), erosion (malignancy, ulcerative disease), and physical disruption (trauma, iatrogenic injury), which is the etiology in the present case.

Perioperative antibiotic administration is essential in the management of intestinal trauma to reduce the risk of infectious complications. Although culture-directed therapy is preferred, the unpredictable nature of abdominal injuries often necessitates the early use of empiric broad-spectrum antibiotics, particularly when the extent of injury is uncertain [9].

The Eastern Association for the Surgery of Trauma (EAST) provides clear recommendations to guide clinical practice. According to their level I guidelines, a 24-h course of prophylactic antibiotics is sufficient following the surgical repair of intestinal trauma, provided there are no complicating factors. However, in scenarios where delayed presentation has led to the onset of an intra-abdominal infection, the guidelines advocate for a four-day course of antibiotics following definitive source control.

Exploratory laparotomy in trauma cases follows a systematic approach [10]. The procedure involves the following four essential stages: control of hemorrhage to arrest bleeding, control of contamination to prevent infection, diagnosis of all injuries to identify visible and hidden damage, and reconstruction to repair and restore anatomical integrity. This method ensures prompt and effective trauma management. Evaluating intestinal injuries as follows: once hemostasis is achieved, assess the small bowel from the ligament of Treitz to the ileocecal junction, controlling any spillage. Inspect the colon, rectum, and mobilize the stomach to check for injuries.

Repair options for intestinal trauma vary based on the severity and context of the injury [11]. Primary repair is suitable for partial-thickness or small full-thickness injuries, such as those less than 3 cm in the stomach or involving less than 50% of the intestinal circumference. Resection and anastomosis are indicated for larger injuries or multiple closely situated injuries, using either staplers or hand-sewn techniques. Diversion is typically avoided in low-risk patients but may be necessary for high-risk cases or when bowel continuity cannot be restored within 36 h, leading to gross contamination or peritonitis. The damage control approach prioritizes patient stabilization in cases of acidosis, hypothermia, or massive transfusion, with definitive repair performed within 36 h once the patient's condition permits.

Primary bowel anastomosis is generally a safe option in cases of intestinal perforation. However, hemodynamic instability requiring pressor support serves as a contraindication, warranting a damage control approach instead. In such cases, leaving the patient in a state of discontinuity is a reasonable interim measure, with a planned “second-look” operation performed soon after to perform the anastomosis and achieve abdominal wall closure [8]. This staged strategy prioritizes patient stabilization while ensuring definitive repair in a controlled manner.

## Conclusions

Traumatic gastrointestinal injuries, increasingly associated with road traffic accidents and workplace hazards, have shown a rising prevalence, particularly among adult males, who represent a higher-risk demographic. Perforations of the gastrointestinal tract most commonly affect the jejunum, owing to its anatomical susceptibility, and are typically managed through primary closure to restore bowel integrity. In cases of suspected workplace injuries, obtaining a comprehensive occupational history is crucial. This includes details about the patient's employment, specific job tasks, and exposures to hazardous equipment or environments. Such information not only aids in diagnosing the underlying trauma mechanism but also ensures tailored management strategies to address potential complications effectively. Preventive measures, such as closed enclosures around the drilling machine and mining area, along with strict prohibition of human interference within the surrounding area, can easily prevent such injuries. Education of concerned staff, miners, and laborers about such injuries is of utmost importance.

Early diagnosis and treatment lead to better outcomes and a better prognosis. Resection and anastomosis were done in the present case. Computed tomography is done in stable cases to determine the location and number of foreign bodies. Timely surgical intervention is crucial and is strongly associated with favorable recovery outcomes in cases of gastrointestinal trauma. Conversely, delays in diagnosis and management significantly increase the risk of complications, leading to higher morbidity and mortality rates. Early surgery not only minimizes the progression of tissue damage and infection but also enhances the chances of optimal functional recovery. Therefore, prompt evaluation and decisive action remain fundamental to improving patient prognosis in such critical conditions.
